# Covering Chemical Diversity of Genetically-Modified Tomatoes Using Metabolomics for Objective Substantial Equivalence Assessment

**DOI:** 10.1371/journal.pone.0016989

**Published:** 2011-02-16

**Authors:** Miyako Kusano, Henning Redestig, Tadayoshi Hirai, Akira Oikawa, Fumio Matsuda, Atsushi Fukushima, Masanori Arita, Shin Watanabe, Megumu Yano, Kyoko Hiwasa-Tanase, Hiroshi Ezura, Kazuki Saito

**Affiliations:** 1 RIKEN Plant Science Center, Yokohama, Japan; 2 Graduate School of Life and Environmental Sciences, Gene Research Center, University of Tsukuba, Tsukuba, Japan; 3 Department of Biophysics and Biochemistry, The University of Tokyo, Tokyo, Japan; 4 Graduate School of Pharmaceutical Sciences, Chiba University, Chiba, Japan; Cinvestav, Mexico

## Abstract

As metabolomics can provide a biochemical snapshot of an organism's phenotype it is a promising approach for charting the unintended effects of genetic modification. A critical obstacle for this application is the inherently limited metabolomic coverage of any single analytical platform. We propose using multiple analytical platforms for the direct acquisition of an interpretable data set of estimable chemical diversity. As an example, we report an application of our multi-platform approach that assesses the substantial equivalence of tomatoes over-expressing the taste-modifying protein miraculin. In combination, the chosen platforms detected compounds that represent 86% of the estimated chemical diversity of the metabolites listed in the LycoCyc database. Following a proof-of-safety approach, we show that 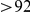
% had an acceptable range of variation while simultaneously indicating a reproducible transformation-related metabolic signature. We conclude that multi-platform metabolomics is an approach that is both sensitive and robust and that it constitutes a good starting point for characterizing genetically modified organisms.

## Introduction

The internationally accepted substantial equivalence (SE) framework [1], [2] proposes to begin risk assessment of genetically-modified (GM) organisms by comparing them with traditional varieties. Only compounds not present in similar amounts in already accepted traditional varieties need to be subjected to toxicological testing [3]. The goal of SE evaluation is not to draw a conclusion about the novel organism's safety status because that would require the impossible testing of all compounds. Instead, by examining a broad set of traits, SE evaluations aim at obtaining a picture of the magnitude and nature of incurred changes to use as a screen for potentially problematic changes and a starting point for further investigations [3].

As omics strategies are applied to measure as many features of the target system as possible, they are a natural choice for evaluating SE. Reported applications include transcriptomics [4], [5], proteomics [6], and metabolomics [7]–[10]. Of these, metabolomics is of particular interest because the composition of low-molecular-weight molecules is closely related to the organism's phenotype and includes important nutritional and toxicological characteristics [2], [11].

An often undervalued issue in applications of metabolomics for SE is that the set of profiled metabolites must be sufficiently diverse and representative to permit a general conclusion about the SE status. However, currently there is no technique that can achieve the complete separation of all types of molecules [12]. A combination of separation-free fingerprinting [13], [14] followed by focused profiling of regions with strong differences has been proposed to address this question [8], [9], [15]. However, a conceptual problem with fingerprinting is that although the profiles are derived from the whole sample, no metabolites are identified. Consequently, the detection performance cannot be evaluated empirically and an objective estimate of the SE status becomes difficult to obtain. Here we propose to reduce the chemical bias by acquiring data from a combination of untargeted gas chromatography- (GC), liquid chromatography-quadrupole (LC-q), and capillary electrophoresis (CE)-time-of-flight (TOF) mass spectrometry (MS). All the chosen platforms allow for metabolite identification using standard libraries and the resulting data can therefore be directly interpreted and evaluated in terms of the performance in detecting chemically diverse metabolites. In order to facilitate analysis and interpretation we combine the data to a single consensus data set. Briefly, this is achieved by performing automated data format and metabolite identifier unification [16], followed by summarization of multiple measurements of the same metabolites using principal component analysis (PCA).

Another point of underestimated value in omics-based SE assessments is the question of how to quantify the evidence for similarities between the novel and the control organism. The central strategy in previously reported studies (e.g. [10], [17], [18]) has been to perform ordinary ANOVA to identify differences; lack thereof has been interpreted as evidence for sufficient similarity. However, lack of an effect can not be shown in this manner since “absence of evidence is not evidence of absence” [19]. Instead, it is necessary to use a proof-of-safety approach where non-similarity is used as the null-hypothesis, and similarity the alternative [20], [21]. In quantitative targeted settings, this may be achieved by using dual ANOVA to test for levels exceeding externally defined acceptable upper and lower limits. In untargeted semi-quantitative assays such as metabolomics, such limits are not available. In order to quantify the similarities also in this scenario, we propose to use a panel of traditional cultivars to dynamically define the borders of the null-hypothesis as the estimated levels of the cultivar farthest away from the control line. Rejection of the proof-of-safety null-hypothesis may then be interpreted as an indication of acceptable metabolite levels. Still, metabolomics measurements are inevitably affected by technical factors such as matrix effects and ion suppression; this weakens the chain of evidence. To avoid this problem we furthermore propose the testing of only peaks that respond to the experimental design in a predictable manner. In this way we obtain evidence that the biological variance is well-detected, thereby arriving at an objective SE assessment.

Hand in hand with the efforts to narrow down the list of potentially problematic metabolites, it is important to identify those that are differentially abundant — whether within acceptable limits or not — as these may provide insight in the physiological status of the novel organism. Depending on its nature, such information may serve as a guide for the development of future similar lines, cultivation and product usage.

Interpreting a large number of hypothesis tests when the magnitude of the expected changes is small is difficult due to an unavoidable large proportion of false positives. Complementary to ANOVA, we therefore use orthogonal projections to latent structures discriminant analysis (OPLS-DA) models [22] to obtain lists of the most influential metabolites that also can be compared across different experiments.

The goals of this study can be summarized as i) establishment of a multi-platform metabolomics approach for SE evaluations including an assessment of the achieved coverage of the chemical diversity; ii) development of a data analysis strategy to both screen for potentially problematic metabolites (using the proof-of-safety approach) and to detect transformation related changes; iii) to provide a case-study of the proposed work-flow. To meet the last goal, we evaluated the metabolomic SE status of tomatoes that over-express miraculin [23], a glycoprotein with the remarkable ability to change a sour- into a sweet taste. This makes it a potential low caloric natural sweetener and flavor enhancer. The source of the miraculin gene, *Richadella dulcifica*, is a tropical plant that is difficult to grow outside its natural habitat. Therefore, efforts have been made to express miraculin in other organisms [24], [25].

Defining non-similarity as a greater deviation from the control cultivar (Moneymaker) than a threshold decided by the traditional reference cultivars, we found evidence of acceptable metabolite levels for 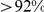 of the evaluated peaks and list the remaining peaks as potential subjects for further inspection. OPLS-DA models of data from two independent experiments revealed a slight reduction in asparagine levels and an increase in proline and spermidine levels as potential unintended effects of genetic modification.

## Results

### Multi-platform metabolomics work-flow for evaluating SE

**Figure 1:**
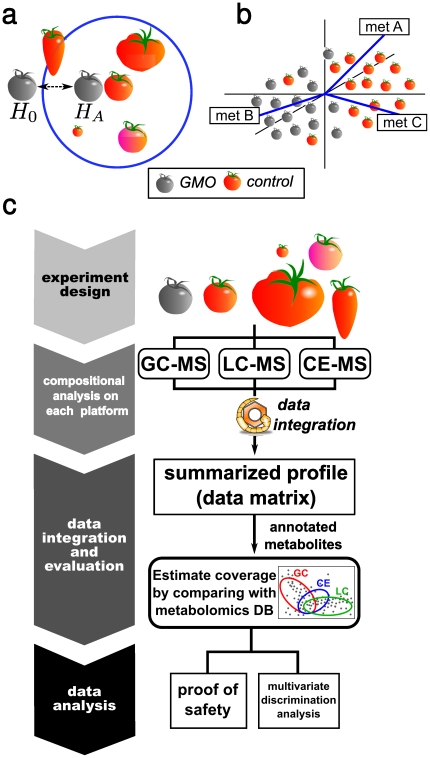
A multi-platform metabolomics approach for evaluating SE. (a) The first task is to quantify the evidence for a safe molecular composition. This is done by testing the null-hypothesis (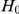), which states that the genetically modified organism (GMO) deviates more from the control line than a panel of traditional cultivars, against the alternative hypothesis of SE (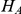). (b) The second task is to look for discriminative features [e.g. metabolite (met) A, met B and met C] between the transgenic line and the control to obtain an understanding of the consequences of the incurred effects. (c) The proposed work-flow. Samples are analyzed on three analytical platforms. The resulting data sets are summarized to consensus, non-redundant data sets with the help of the MetMask metabolite identifier management tool [16]. The achieved coverage is evaluated by comparing the chemical properties of the detected metabolites with a reference metabolome in the literature. A proof-of-safety approach is used to quantify the evidence for safe metabolite levels; multivariate discrimination analysis is used to characterize the unintended effects.

We assessed the metabolomic SE status of the genetically-modified organisms from two perspectives. First we aimed at establishing the ratio of all metabolites that objectively can be considered to be within acceptable ranges of variation (Figure 1a). Next we attempted to characterize the nature of GM related incurred changes and to obtain a picture of their physiological consequences (Figure 1b). With the workflow outlined in Figure 1c we analyzed the metabolomic profiles of the transgenic- and the control lines and of a panel of traditional cultivars.

The magnitude of the required experiments and our demand for wide metabolomic coverage placed high demands on the analytical platforms in terms of robustness and metabolite identification capabilities. Among current analytical techniques, time-of-flight (TOF)/MS is a particularly suitable detection system as it combines high sensitivity and spectral resolution with a broad mass range and high throughput. For the detection of primary- and polar secondary metabolites and of ionic compounds we proposed to use three untargeted TOF/MS-based platforms, i.e. GC-TOF/MS (GC-MS), LC-q-TOF/MS (LC-MS), and CE-TOF/MS (CE-MS), respectively. These three platforms generated separate data sets that we fused using a novel data summarization strategy [16] (Figure 1c, middle). The achieved coverage can then be evaluated by comparison with a reference metabolic pathway database.

The analysis of the obtained data is divided into two related but from a statisticians point of view distinctly different concepts. In the first step, we employ the proof-of-safety approach [21] to test for acceptable deviation(s) from the control using the panel of traditional cultivars to define acceptable ranges of variation. Here, we screen for conspicuous metabolites that may require further evaluation but do not address the existence of significant differences *per se*; this is addressed in the following step. Using OPLS-DA [22] we construct models of the differences between the transgenic- and the control lines. The goal here is to obtain an understanding of the consequences of the genetic modification. OPLS-DA was chosen as it permits direct extraction of genotype-related variances even in the presence of uncontrolled co-variates or factors whose exact definition may be difficult, for example, the ripening stage.

In the following sections we describe an application of this strategy; we report an SE evaluation of two independent lines of miraculin over-expressing tomato (*Solanum lycopersicum*, L. cv. Moneymaker), 56B and 7C.

### The use of multiple platforms improves coverage of the tomato metabolome

**Figure 2:**
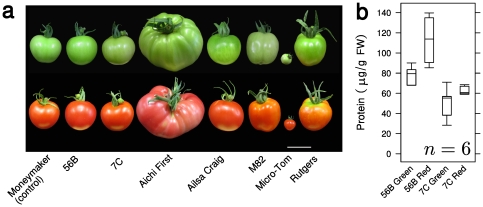
Tomatoes grown on hydroponic culture (HC) solution. (a) Visible phenotypes of the transgenic lines (56B and 7C), the control line Moneymaker, and five reference cultivars. The scale-bar represents 5 cm. (b) Miraculin protein accumulation in the two transgenic lines harvested in green and red stages. The protein levels were determined by enzyme-linked immunosorbent assay (ELISA). The horizontal lines in the boxes correspond to distribution quartiles.

The physiological status of plants is highly dependent on their developmental stage and on nutritional and environmental conditions. The two most common methods of tomato production are cultivation on hydroponic culture (HC) solution and on soil; we performed a pilot experiment to estimate the differences in the metabolism and miraculin production under the two growth conditions. The effect of varying these conditions on metabolite levels was small but significant (Figure S1 in File S1). Miraculin levels indicated higher protein accumulation on HC solution (Figure S2 in File S1). We posited that the magnitude of unintended effects attributable to the expression of miraculin increases with the amount of accumulated miraculin. Therefore, we performed a large-scale experiment on HC solution in which we compared two transgenic- with the control line and five reference cultivars; harvesting was done in their green and red ripening stages. The phenotype of the transgenic fruits exhibited no visible differences compared to the control line (Figure 2a). Miraculin accumulation was confirmed to be high and stable in both ripening stages in the two transgenic lines (Figure 2b).

The summarized data from all three platforms include 175 unique identified metabolites and 1460 peaks with no or imprecise metabolite annotation. Of the identified metabolites, 56 were detected on more than one platform and these showed an average pair-wise cross-platform correlation of 0.50 (Figure S3 in File S1).

**Figure 3:**
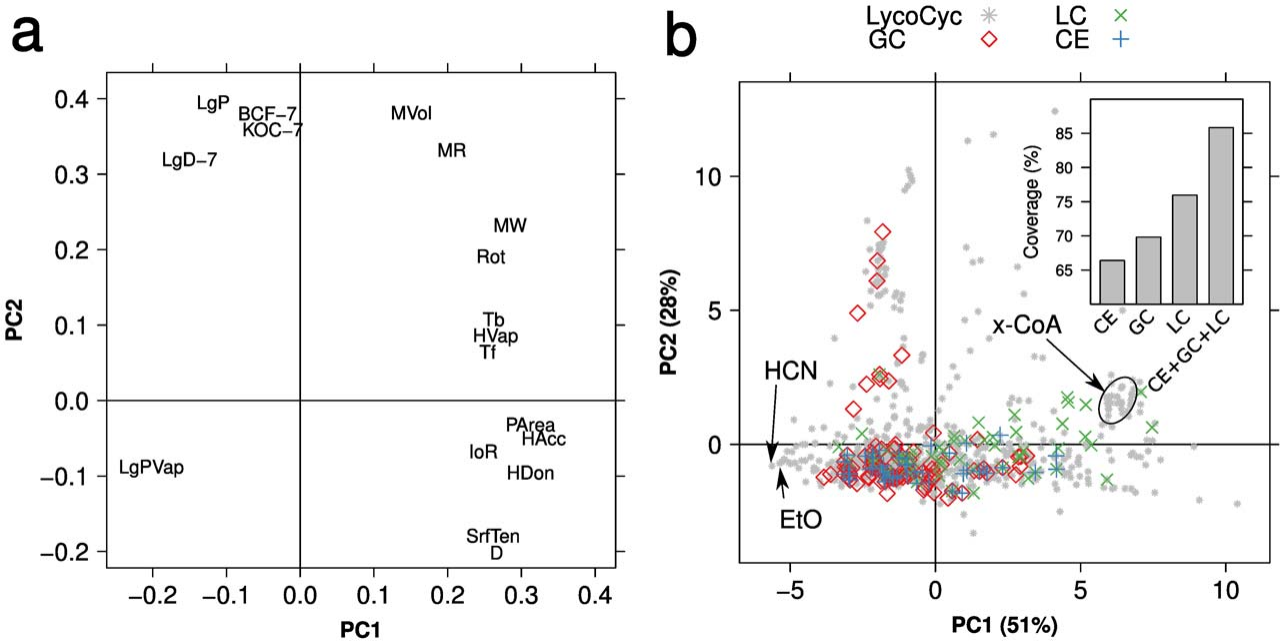
Evaluation of the achieved coverage. PCA was performed on the predicted physicochemical properties of the detected metabolites and the metabolites in the LycoCyc database. (a) The loading plots show that PC1 is dominated by size-related- and PC2 by solubility-related properties. (b) The score plots show that the distribution of the detected metabolites occupies a similar space as the reference metabolites. The inset barplot shows the ratio of variance among the reference metabolites covered by each of the individual platforms and the summarized data set. No small volatile molecules such as hydrogen cyanide (HCN) and ethyl aldehyde (EtO) or large secondary metabolites represented by the cluster of CoA ligates (x-CoA) were detected. Abbreviations: Log vapor pressure (LgPVap), octanol: water partitioning coefficient (LgP), octanol:water solubility distribution coefficient at pH 7.4 (LgD-7), biological concentration factor at pH 7.4 (BCF-7), adsorption coefficient at pH 7.4 (KOC), molecular volume (MVol), molecular refractivity (MR), molecular weight (MW), free rotating bonds (Rot), boiling temperature (Tb), flash point (Tf), enthalpy of vaporization (HVap), polar surface area (PArea), number of H-bond donors/acceptors (HDon/HAcc), surface tension (SrfTen), density (D), index of refraction (IoR).

To evaluate the detection performance, we extracted 816 metabolites from the tomato metabolism database LycoCyc (http://solgenomics.net/tools/solcyc/) [26] to use as a reference. Only 55 of the detected metabolites overlapped with LycoCyc, indicating incompleteness of the LycoCyc database, difficulties arising from the high number of similar but not identical metabolites in plant metabolism, and the tissue dependency of metabolite occurrences. Therefore, instead of carrying out direct comparisons we used physicochemical properties of the two sets of metabolites as a proxy to compare their chemical diversities. We used 18 features that could be obtained for 160 of the detected annotated metabolites and for 658 of the 816 LycoCyc metabolites. Principal component analysis (PCA) of the combined data showed good overlapping of the distribution of the detected- and the LycoCyc metabolites (Figure 3a-b). As expected, GC-MS mainly detected low molecular weight compounds including carbohydrates and amino- and organic acids whereas LC-MS excelled in the detection of heavier molecules with a larger polar surface area (e.g. flavonoids). Compounds detected by CE-MS were distributed among LC-MS and GC-MS compounds. Areas that were not covered are exemplified by the cluster of large CoA-ligates and the small, volatile molecules hydrogen cyanide and ethyl aldehyde.

Using separate PCA models for the metabolites in the summarized data set and those from the individual platforms respectively, we calculated how well the different subsets of metabolites approximate the total chemical diversity (variance in the physicochemical properties) of LycoCyc (see Materials and methods). The PCA model of all detected metabolites accounted for 86% of the chemical diversity which represents a wider coverage than was achieved with any of the platforms individually, indicating that they are complementary (inset barplot in Figure 3b).

### Miraculin over-expressors are remarkably similar to the control line

**Figure 4:**
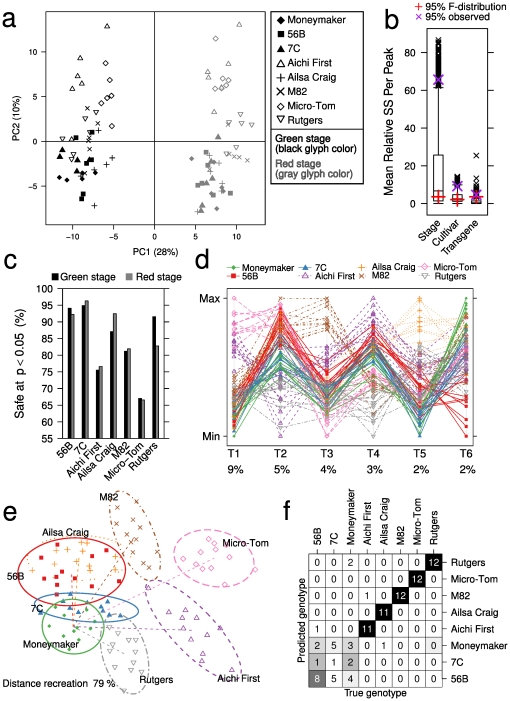
Evaluating SE of miraculin over-expressing tomatoes grown on HC solution. (a) Score scatter plot of PCA of the annotated metabolite profiles. Separation of the two ripening stages can be seen on PC1 and of the different cultivars on PC2. (b) Contribution to variance of the different experimental factors. Each peak was scaled to a total sum of squares (SS) of 100. Peaks above the 95th F-distribution percentile indicate significance at 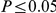. Factors with separated observed- and F-distribution percentiles indicate the overall significance of that factor. (c) The ratio of peaks considered to indicate safety at a significance level of 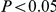 compared to the Moneymaker line. For comparison purposes, the test was applied to the transgenic lines and the traditional cultivars. (d) Parallel coordinates plot of the predictive components from the OPLS-DA model. Each biological sample is drawn as a line that connects its positions on each of the components. Each dimension describes a unique aspect of the genotype-correlated variance among the metabolite profiles. All genotypes except 7C and Moneymaker are separated on at least one axis. Percentages indicate the ratio of total variance explained by the corresponding dimension. (e) Result from Sammon's MDS of distances computed using the six predictive OPLS-DA components shown in (d). (f) Confusion matrix for predicting the genotype using the OPLS-DA model during five-fold cross-validation.

We performed PCA to obtain an overview of the annotated summarized data set (Figure 4a; loadings are listed in Data S1 in File S2 and PCA of the complete data set is shown in Figure S4a in File S1). The score scatter plot indicates that the main sources of variance were related to the ripening stages (PC1) and the cultivars (PC2) rather than the transgenic status of the plants. The same conclusion can be drawn from ANOVA results (see the overview of variance contribution in Figure 4b). Note that this result does not preclude differences between the transgenic lines and Moneymaker, but rather indicates that if they exist, then they are smaller than the differences between ripening stages and different traditional cultivars.

In the complete data set, 1376 of the total 1461 peaks (84%) showed a significant correlation with the experimental factors genotype and ripening stage. The source of variation among the remaining 261 peaks could not be reliably determined. As this rendered their analytical accuracy unclear we excluded them from subsequent proof-of-safety analysis. The remaining data contained 166 identified metabolites with 85% coverage of the chemical diversity of LycoCyc.

Defining acceptable deviation as being within the symmetric boundary decided by the traditional cultivar furthest away from the control line, we performed a proof-of-safety analysis for the metabolite levels of the transgenic lines. The null hypothesis that the transgenic lines are outside this boundary could be rejected for 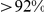 of the 1376 tested peaks for both transgenic lines 56B and 7C (Figure 4c). The proof-of-safety test was inconclusive (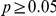) for 310 of the tested peaks in at least one transgenic lines and ripening stage and these peaks are listed Data S2 in File S2. The average fold-change over the control line was lower or similar to the accepted upper limit for all of these peaks. This indicates that the majority of them receive high p-values due to strong variance rather than clearly being outside the accepted thresholds. As a comparison, we performed the same analysis for the traditional cultivars by treating them as hypothetical transgenics with unknown safety status (Figure 4c). The ratio of peaks that passed the proof-of-safety test ranged between 67 (Micro-Tom, red stage) and 92% (Ailsa Craig, red stage). The result from the proof-of-safety analysis depends on the definition of acceptable deviation. Here we present a direct adaptation of the procedure outlined in ref. [21]. See Text S1, Section 1.3, in File S1 for a detailed description and the results obtained using asymmetric thresholds for acceptable deviation in Figure S4b in File S1.

### Identifying unintended GM effects

After screening for potentially problematic metabolites and identifying those for which there is evidence of safety, we addressed the nature and magnitude of the incurred differences. We first fitted an OPLS-DA model of all samples to obtain an overview of genotype-dependent variances. Cross-validation pointed to six predictive components that together associated 25% of the variance with the genotype. They are shown in Figure 4d as a parallel coordinates plot. To obtain a better overview of the distances between the different cultivars we computed the pairwise Euclidean distances between all observations using only the six predictive components. The distance matrix was then compressed into two dimensions using Sammon's multi-dimensional scaling (MDS) [27]. Figure 4e presents the obtained visualization with each cultivar encircled by a 95% confidence ellipse. The distances within- are typically smaller than between genotypes, except for an apparent confusion among Moneymaker, the transgenic lines, and Ailsa Craig. Five-fold cross-validation showed that Ailsa Craig could actually be well recognized, but the controls and the two transgenic lines were internally mixed up (Figure 4f). The miraculin over-expressing lines are thus closer to the control line than any of the traditional cultivars.

### Two independent experiments indicate reproducible unintended effects of GM

As we could not detect conclusive differences between the control- and transgenic lines despite the high miraculin accumulation in the latter (Data S3 in File S2), we performed focused experiments using only the control- and transgenic lines. The growth medium was changed to soil and two watering regimes were applied to monitor the interactions under different watering conditions. The fruits were sampled in their red ripening stage.

Miraculin protein accumulation was lower when both lines were grown on soil than on HC solution; the average was 11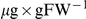 for 7C and a mere 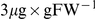 for 56B. The miraculin mRNA levels showed a similar trend (Figure S2 in File S1).

Multi-platform metabolite profiling resulted in a summarized data set with 120 unique annotated metabolites and a total of 1033 peaks. Twenty-six metabolites were identified on more than one platform with the average pairwise cross-platform correlation 0.47 (Figure S3 in File S1).

PCA and ANOVA indicated that the largest source of variation was the difference in harvesting time (Figure S5a-b in File S1). The plants' genotype and watering treatment accounted for similar ratios in total variance but showed no significant interaction effect. By ANOVA (Modified 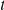-test [28]), 113 and 80 peaks were significantly different between Moneymaker and lines 56B and 7C, respectively (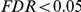). Data S4 in File S2 lists the annotated metabolites that were differentially abundant in both 56B and 7C. We observed no significant correlation between the metabolite levels and miraculin accumulation looking at the 7C samples (see the comparison between observed 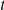-statistics and the 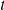-distribution under the null-hypothesis in Figure S6 in File S1).

**Figure 5:**
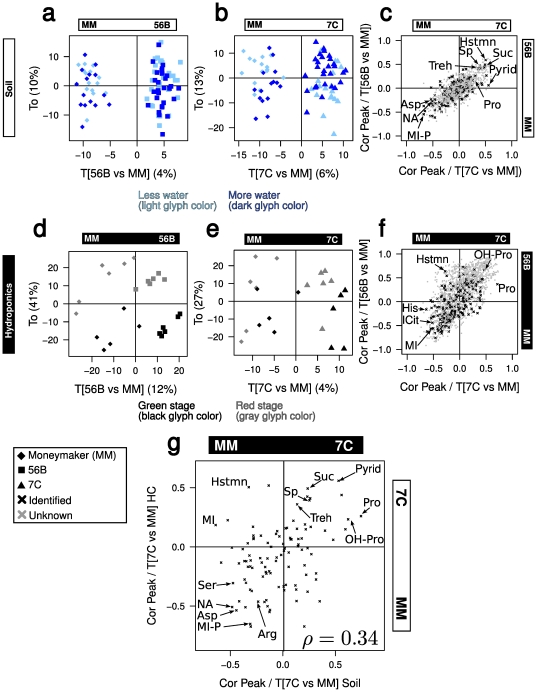
Focused OPLS-DA models for discriminating the Moneymaker (MM)- from the transgenic lines. Percentages on the axes indicate the ratio between the explained and the total variance. The predictive components 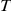 (56B and 7C) are correlated to the genotype; the 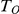 components (56B and 7C) are orthogonal. (a–b) Score plot for the OPLS-DA model between 56B and MM using data from the soil experiment and (b) 7C and MM (b). (c) Correlation loading plots show the well-described peaks. The correlation indicates an overlap between the metabolites that are used to isolate 56B and 7C. The two models associate 4% and 6% of the variance to the genetic modification of 56B and 7C, respectively. (d–f) OPLS-DA models using the metabolite profiles of tomatoes grown on HC solution. (g) Overlap between the metabolites used to discriminate 7C and Moneymaker using metabolite profiles from soil and HC experiments. Asparagine (Asp) levels are lower in 7C than MM and the proline (Pro) levels are higher. Metabolite abbreviations are shown in Data S5 in File S2.

OPLS-DA could separate both 56B and 7C from the control with near perfect accuracy by cross-validation; however, it associated only 4 and 6% of the variance with the genotype correlated component 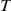 (Figure 5a-b). The empirical 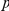-values from resampling tests were strongly significant (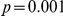 for both 56B and 7C). The genotype-unrelated components explained approximately 10% and 13%, respectively, of the variance and was strongly correlated with the chronology of harvesting (Spearman's 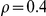 [56B] and 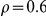 [7C], Figure S7a in File S1). The correlation loadings, the proximity between each peak and the predictive component, showed a clear overlap between the two models (Figure 5c) with, for example, increased spermidine- and decreased inositol-1-phosphate levels.

To compare these results across the used growth conditions we performed the same focused OPLS-DA for 56B and 7C versus the control using the HC data (Figure 5d-e). The prediction accuracy of these models was low but greater than with randomized data, the empirical p-values from resampling tests (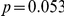, 7C and 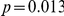, 56B). Similar to the models from the soil experiment, the HC-based models for 56B and 7C showed an overlap in the correlation loadings. Here the transgenic lines had higher levels of 4-hydroxy-proline and proline and lower levels of myo-inositol than the control (Figure 5f).

Comparison of the loadings of the commonly-detected metabolites between the HC and the soil experiment showed a correlation that was significantly greater than zero for the two independent models of 7C (Spearman's 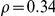, 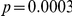, 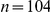), but not the models of 56B (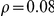, Figure S7b in File S1). Figure 5g is a scatter plot between the two loading vectors for the 104 metabolites that were identified in both experiments and other metabolites indicated on the Y and X axes. Proline, 4-hydroxy-proline, and spermidine manifested relatively high loadings under both conditions, indicating that their levels were higher in 7C than the control. On the other hand, asparagine, arginine, serine, and inositol-1-phosphate were less abundant.

## Discussion

Untargeted metabolomics is like casting a net over all metabolites; it facilitates the broad and unbiased screening of an organismâ€™s molecular composition. The goal of an SE evaluation is not only the identification of unintended changes but also the quantification of evidence for overall similarity. As such evidence can only be obtained for actually detected compounds the question of safety cannot be answered fully: the unacceptable accumulation of an undetected metabolite(s) cannot be ruled out.

Therefore, the usefulness of an untargeted SE evaluation depends on having an assessment of the profiling performance — the size of the net and the coarseness of its mesh — to estimate the likelihood of such a rogue metabolite(s). In the present study we combined GC-MS, LC-MS, and CE-MS to profile transgenic tomatoes. These platforms support metabolite identification and using a strategy from the field of drug discovery [29], we showed that the platforms are complementary. The reliably detected metabolites together approximated 85% of the chemical diversity seen in the LycoCyc database, LC-MS alone captured 76%, GC-MS 70% and CE-MS 65% (Figure 3b). A larger percentage indicates a better coverage but care should be taken when interpreting it quantitatively since different metabolite classes exhibit different ranges of diversity in their physicochemical properties. LC-MS focuses on secondary metabolites which are very diverse in a wide range of properties (e.g. size, density and vapor pressure). GC-MS and CE-MS on the other hand mainly detect primary metabolites which are relatively homogenous compared to the secondary metabolites. For the purpose of an unbiased evaluation, all types of metabolites are of interest and even small increases in coverage is therefor desirable. The evaluation of metabolomic coverage presented here would be improved by using a much broader set of physiocochemical properties and this will be a topic for future studies in our research group.

We emphasize that the introduced coverage statistic does not serve to estimate the total metabolomic coverage; this can only be estimated given a list of all available metabolites, but such a list arguably very difficult to create. Instead, our analysis serves to indicate that any abundant but undetected metabolite is likely to exhibit exotic properties compared to the known tomato metabolome, or to be similar to the types of molecules that we did not detect e.g. small volatiles and very large secondary metabolites. Customizing the protocols to facilitate the detection of such molecules could be a next step in improving chemical coverage.

The reproducibility between different platforms (Figure S3 in File S1), the overlap of ripening-related changes with previous studies [30], [31] (Figure 4a, Data S1 in File S2), and earlier validations using external standards [32], [33] indicate good overall analytical precision of the platforms we used. To control for satisfactory precision with respect to individual metabolites, we applied a test to ensure that the data correlated well with known experimental factors. With this approach we obtained evidence that any undetected differences attributable to GM are smaller than are the differences between different cultivars and ripening stages.

The first step in our SE evaluation was proof-of-safety analysis; it showed that 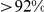 of the tested peaks (Figure 4c) deviated less from the control line than the accepted limit estimated using the reference panel of traditional cultivars. The inconclusive peaks showed relatively small changes with all averages being below the accepted upper limit. This indicates that high variance, rather than a shift in the average, was the predominant reason for failing the proof-of-safety. That said, the list of metabolites that did not pass the test (Data S2 in File S2) may provide a guide for designing future quantitative targeted analysis.

In the second step we used OPLS-DA to look for changes attributable to GM. Discrimination analysis using the metabolite profiles from the HC experiment indicated a high overall proximity between the transgenic lines and the control (Figure 4e). A possible reason for the modest phenotypical differences is that miraculin is xenogenic and presumably metabolically inert in tomato. In addition, it is exported from the cell [23], [34] and this may further limit metabolic interference.

The small impact of GM was confirmed in the soil experiment where only 4–6% of the variance was contributed by differences between the genotypes; the harvesting index accounted for nearly twice as much. The relatively very low variance associated with GM concurs with large scale data of GM maize and soybean [35].

Interestingly, the changes found in 56B, and 7C were similar in both experiments (Figure 5c,f) although 56B accumulated almost no miraculin when grown on soil. An explanation for this could be that the differences between transgenic lines and the control are due both to over-expression-related- and non-related pleiotropic effects. Contributing factors to the pleiotropic effect could be heritable epigenetic regulation attributable to tissue culturing, the transformation procedure [36]–[38], the position of the insert, and the marker gene used for selection. Hypothesizing that the effects are additive, we expected higher miraculin accumulation to result in a stronger deviation of 56B than 7C from the control; the discrimination analysis supports this hypothesis (Figure 5d-e). On soil, 56B accumulated almost no miraculin; the differences from the control were dominated by the miraculin-unrelated effect. On the other hand, 7C accumulated more miraculin (Figure S2 in File S1) and therefore exhibited both effects. Consequently, we observed overlapping between the HC- and the soil experiments for 7C (Figure 5g). The correlation between the loadings from the independent HC and soil based models for 7C highlights that multivariate approaches are better at finding small concerted changes among a large number of variables than corresponding univariate approaches.

The hypothesized miraculin-related metabolic signature is characterized by a decrease in asparagine which is involved in the nitrogen metabolism during the ripening stages [30], an increase in the reliable stress indicator proline, and the anabolic growth regulator spermidine [39]. Furthermore, the levels of inositol and its precursor inositol-1-phosphate were decreased. The inositol levels have been shown to change during ripening [40] and to vary across different varieties [41] of tomatoes.

Taken together, our findings lead us to conclude that our multi-platform approach yields a wide and robust characterization of the tomato-fruit metabolome. The differences between the transgenic lines and the control were small compared to the differences observed between ripening stages and traditional cultivars. The next step in SE evaluation may focus on other types of molecules such as large secondary metabolites and proteins.

## Materials and Methods

### Plant material

Metabolomics meta data formatted according to guidelines of The Metabolomics Standards Initiative [42] is given in Text S2 in File S1. Two miraculin over-expressing tomato lines, 56B and 7C (*Solanum lycopersicum* L. cv. Moneymaker) [23], and the traditional cultivars Moneymaker, Aichi First, Ailsa Craig, Micro-Tom, M82 and Rutgers were grown in a netted-greenhouse at University of Tsukuba in 2006, 2008 and 2009. Both transgenics had single inserts of the miraculin gene (Figure S8 in File S1) with shown stable inhertiance to T5 [43]. Three experiments were performed: a pilot experiment using fruits of the Moneymaker, 56B and 7C at the red stage grown on soil and HC with harvest in spring, a large scale experiment on HC using all genotypes and both green and red ripening stages (stage determined visually) also harvested in spring, and an experiment with fruits of the Moneymaker, 56B and 7C at the read stage grown on soil with harvest in late summer (Table S1 in File S1). In the soil experiment, plants were grown under high-watered or low-watered conditions controlled by an automatic water supply device (UNSU CSK-5500, Sankeiriika inc.). The amount of the diluted Hyponex solution (N-P-K = 6-10-5: EC1., HYPONeX JAPAN Co., Ltd.) supply was determined by soil water potential values. Miraculin mRNA expression and protein accumulation was measured using quantitative real-time polymerase chain reaction (qRT-PCR) and enzyme-linked immunosorbent assay (ELISA) respectively as described in ref. [25], [44]. The harvested fruits were chopped and 1 g fresh weight (FW) of the pieces was put in a 2-ml tube with 5 mm Zirconia beads to be used for metabolomics profiling and 3 g was saved for ELISA and qRT-PCR assays. The frozen samples were lyophilized before metabolite profiling.

To compare these results across the used growth conditions we performed the same focused OPLS-DA for 56B and 7C versus the control using the HC data (Figure 5d-e). The prediction accuracy of these models was low but greater than with randomized data, the empirical p-values from resampling tests (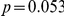, 7C and 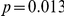, 56B). Similar to the models from the soil experiment, the HC-based models for 56B and 7C showed an overlap in the correlation loadings. Here the transgenic lines had higher levels of 4-hydroxy-proline and proline and lower levels of myo-inositol than the control (Figure 5f).

### Metabolite profiling

All data was 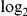 transformed and scaled to unit-variance prior to further data analysis. All peaks with more than 30% missing values were excluded. The detected metabolites are listed in Data S5 in File S2. The final summarized data sets are available at http://prime.psc.riken.jp/?action=drop_index.

**GC-MS** was performed as described in ref. [45]. A total of 0.5 mg dry weight (DW) of the fruit samples were subjected to derivatization and an equivalent of 0.6 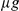 and 6 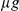 of the derivatized samples were injected into the GC-MS instrument for detection of highly and lowly abundant metabolites respectively. The chromatograms were pre-processed using the HDA method [46] and normalized using the CCMN algorithm [33].

**LC-MS** (negative and positive mode) was performed as described in ref. [47]. Samples were extracted and an equivalence of 125 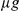 was injected into the instrument.

**CE-MS** (cation and anion mode) was done according to ref. [48]. Measurements were performed using a total of 14 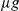 of each sample.

### Data analysis

**Filtering** was done by first removing all peaks with more than 30% missing values. All remaining peaks were then tested for detection performance of biological variance by fitting a linear model between the estimated abundance and first order predictors based on the experimental factors ripening stage, genotype, treatment and harvesting time. Only peaks that could be predicted by this model as decided by ANOVA F-test, 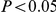, were retained.

**Data summarization** was performed by first unifying platform specific metabolite identifiers to a common non-redundant referencing scheme using the MetMask tool [16]. The three matrices were then concatenated and correlated peaks with the same annotation were replaced by their first principal component to reduce data redundancy. Poorly correlated metabolite-pairs were left as duplicates.

**Coverage of the chemical diversity** was estimated by fetching all available predicted physicochemical properties from the ChemSpider database (http://www.chemspider.com) for the detected metabolites and the metabolites mentioned in the LycoCyc database [26]. Vapor pressure was log transformed and all traits were scaled to unit variance to give them equal importance. Chemical coverage was defined as the percentage of variance among the LycoCyc metabolites that could be predicted using a PCA model of the properties of a given subset of metabolites. Specifically, chemical coverage was defined as:
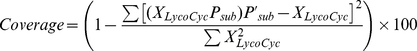
where 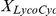 are the unit-variance scaled physicochemical properties of metabolites in LycoCyc, and 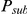 the loadings matrix from the PCA model of the properties of a subset of metabolites (e.g. those from an individual platform). Missing value robust PCA was performed using the pcaMethods package [49].

**Proof of safety analysis** was performed using an adapted version of the method described in ref.[21]. Briefly, acceptable deviations from the control were defined by the symmetric maximum absolute boundaries of the 90% confidence intervals of 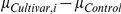 where the 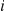
th cultivar is the one furthest away from the control plant (Moneymaker). Safety was declared when the compound null hypotheses stating that the transgene deviate either more or less than the estimated accepted thresholds could be rejected using two one-sided Student's 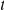-tests with correction for unequal variances. The normality assumption was examined using Kolmogorov-Smirnov (KS) test (Figure S9 in File S1).

**Multivariate discriminant analysis** was performed using OPLS-DA [22]. Briefly, OPLS-DA extracts a set of components, meta features, that describe the class related variance in the metabolite matrix. These components are oriented so that they together discriminate the sought classes well. Another set of components are also calculated that describe as much of the class unrelated variance. The derived model is can be used to predict both the class separating components and the orthogonal components for new data. Resampling tests were done by shuffling the class labels and recomputing prediction accuracy one thousand times, counting the number of occasions where random class labels obtained better or equal accuracy compared to the original labels, 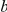; 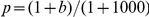.

All data analyses were performed using R v2.12.1 [50]. See Text S1 in File S1 for a more detailed description of the data analysis.

## Supporting Information

File S1 Supporting descriptions of the data analysis, metabolomics meta data as well as supporting Figures S1-S9 and Table S1 and S2.
(PDF)

File S2 Supporting data sets S1-S5. Lists of detect metabolites, metabolite abbreviations, estimated abundance differences.
(XLS)
