# Supplementary material for: Covering Chemical Diversity of Genetically-Modified Tomatoes Using Metabolomics for Objective Substantial Equivalence Assessment
Source: PLoS One. 2011 Feb 16;6(2):e16989. doi: 10.1371/journal.pone.0016989 (PMC3040210; doi:10.1371/journal.pone.0016989)
Supplement: File S1 — Supporting descriptions of the data analysis, metabolomics meta data as well as supporting Figures S1-S9 and Table S1 and S2. (PDF) [file pone.0016989.s001.pdf]

1 Text S1 — Supporting methods

1.1 Data pre-processing

The CE and GC measurements were divided into batches before analysis and the ComBat algorithm [1] was used to suppress the resulting bias. Peaks with more than 30% missing values were dropped. Unannotated small peaks with low reproducibility within biological replicates were also removed.

Data summarization was done by organizing the platform specific data to common formats and unifying metabolite identifiers to a non-redundant referencing scheme using MetMask. Features that carried the same metabolite identifier and were correlated to each other were replaced by their first principal component. This reduced the overall number of missing values and reduced redundancy. Features with same metabolite identifier that were not correlated with each other were left unsummarized.

1.2 Chemical diversity

The coverage of the chemical diversity of the tomato metabolome was estimated by fetching the physicochemical properties predicted by ACD/Labs software at the ChemSpider database for both detected metabolites and all metabolites mentioned in the LycoCyc database. All 22 properties were downloaded where available but polarizability, LogD at pH 5.5, KOC at pH 5.5 and BCF at pH 5.5 was dropped due to redundancy with other features (correlation $\rho > 0.99$).

1.3 Proof of safety

Proof of safety analysis was performed by adapting the method described by [2] section 2.2 to the metabolomics setting. Two one sided two sample Student's t-tests (with correction for non-equal variances) are used to test the null-hypotheses:

$$H_0^+ : \mu_{\text{Transgene}} - \mu_{\text{C}} \geq \delta_+;$$

$$H_0^- : \mu_{\text{Transgene}} - \mu_{\text{C}} \leq \delta_-$$

where δ_+ and δ_- are the acceptable deviations from the control (C). Proof of safety is declared if we can reject both H_0^+ and H_0^- at the chosen confidence level.

For quantitative assays of known compounds δ_- and δ_+ can be estimated using predefined limits dervied from e.g. toxicological studies [2]. For untargeted metabolomics where we also profile unknown compounds, such limits are not available. Assuming that the traditional cultivars deviate from the control cultivar in a safe manner, we estimate acceptable deviation by the upper and lower limits of the t-distribution based 90% confidence intervals, $CI_{0.90}$, for $\mu_{\text{CV},i} - \mu_{\text{C}}$ for the cultivar (CV) furthest away from the control (C):

$$\delta_+ = \arg\max_i \left(\max\left[CI_{0.90,upper}(\mu_{\text{CV},i} - \mu_{\text{C}}); -CI_{0.90,lower}(\mu_{\text{CV},i} - \mu_{\text{C}})\right]\right).$$

Symmetric boundaries were used as described in [2,3] setting $\delta_- = -\delta_+$. This definition estimates the range of the distances between the traditional cultivars and the designated control cultivar.

An alternative definition for the acceptable deviations with assymmetric thresholds was also used and reported in Supporting Figure S 4b. Here we set

$$\delta_+ = \max\left(\arg\max_i \left[CI_{0.90,upper}(\mu_{\text{CV},i} - \mu_{\text{C}})\right]; t_{(1-0.9)/2} s_{\text{C}}/\sqrt{n_{\text{C}}}\right);$$

$$\delta_- = \min\left(\arg\min_i \left[CI_{0.90,lower}(\mu_{\text{CV},i} - \mu_{\text{C}})\right]; -t_{(1-0.9)/2} s_{\text{C}}/\sqrt{n_{\text{C}}}\right).$$

This is a more stringent definition where we only accept actually observed deviances from the control (and the range of metabolite levels observed in the control itself). To evaluate the effect

of relaxing this definition we also report the results of allowing 10 and 20% deviations from δ_- and δ_+. The threshold of 20% is the deviation indicated as acceptable by the Nordic Council of Ministers [2].

Both these tests stands in contrast with the often used null hypothesis

$$H_0^{\Delta} : \mu_{\mathrm{Transgene}} = \mu_{\mathrm{C}}$$

which tests the evidence for difference, i.e. the proof of hazard.

In semi-quantitative measurements we can not easily ensure that the measurements are well correlated with the true concentration differences. Reasoning that all metabolite levels show biological variance across the examined conditions, we test if the assay can capture the biological variation by examining if we can reject

$$H_0^{\mathrm{Noise}} : B = 0,$$

where B is the regression coefficient matrix for the experiment design, X, onto the metabolite levels Y; $Y = XB + E$ using the classical ANOVA F-test. Outlier data were suppressed prior to testing H_0^{Noise} by Winsorizing datum more than three standard deviations away from the median to the maximum or minimum of the measurements within the same limit.

1.4 Multivariate statistics

OPLS-DA was done as described in ref. [4]. Briefly, OPLS-DA extracts a set of components, meta features, that describe the class related variance in X (metabolite matrix with samples as rows and metabolites as columns). These components are given by the matrix T which are discriminate the sought classes well (high correlation with at least one column in the response matrix Y). Another set of components are also calculated that describe as much of the class unrelated variance as possible: the T_O matrix. X is thus decomposed into three separate matrices as:

$$X = TW' + T_O P_O' + E$$

were W and P_O are the weights vector matrices defining the relationship between each variable in X and T and T_O. E is the residual matrix: $E = X - TW' + T_O P_O$. The response matrix Y is a dummy matrix with one column per outcome class with entries corresponding to one for class membership and zero otherwise.

The number of components, n_A and n_{A_0}(columns of T and T_O), the model complexity, are user-defined parameters of the model which we estimate by maximizing the prediction accuracy, a, $((TP + TN)/(TP + TN + FP + FN)$ [true positives (TN), true negatives (TP), false positives (FP), false negatives (FN)] during five-fold cross-validation (CV). All CV segments were balanced to contain similar number of members from each class.

Resampling test for significance of prediction was done by shuffling the rows of the metabolite matrix and calculating accuracy during CV 300 times. The significance p-value then equals the number of equal or better accuracies with shuffled data compared to non-shuffled divided by number of repeats. A total of five different OPLS-DA models were estimated in this study, see Supporting Table S2 for a listing.

PCA was performed using the pcaMethods package [5].

Both PCA and OPLS-DA was performed on unit-variance scaled metabolite matrices to make abundances comparable across metabolites and different platforms.

1.5 Univariate testing of metabolites for differential abundances

In the HC experiment we used the following linear model to test for significant differences between transgenic and control lines for each metabolite m:

$$m = I + g + s + g : s + e \tag{1}$$

where I is the intercept, g is a coefficient describing the genotype (control or transgenic), s describes the ripening stage (red or green), $g : s$ is the interaction term and e the residual. The t-statistic associated with $H_0 : g = 0$ was used to test for significant genotype effect (results reported in Supporting Data 3). For the overview Figure in 4c we used:

$$m = I + c + s + x + e$$

where c indicate the cultivars and x indicates if the sample is coming from a transgene.

In the soil experiment we used the following linear model for each metabolite m:

$$m = I + g + t + h + r + g : t + e \quad (2)$$

where t indicates the watering treatment (more water or less water), h is the harvesting index (numbering 1 for the sample harvested first, 2 for the second and so on), r indicate the truss number of sample and $g : t$ the interaction effect between genotype and treatment, results reported in Supporting Data 4 and Supporting Figure S 5b. Acceptable normality of the distributions of the residuals,e, in (1) and (2) to warrant use of parameteric tests, was examined using the Kolmogorov-Smirnov (KS) test (Supporting Figure S 9).

2 Text S2 — Metabolomics meta data

Meta-data prepared following the current guide lines of the Metabolomics Standards Initiative [6].

2.1 Plant context meta data

2.1.1 BioSource Species

Solanum lycopersicum, L. cv. Moneymaker, Aichi-first, Ailsa Craig, MicroTom, M82, and Rutgers.

2.1.2 Organ

Fruits

2.1.3 Organ specification

Green and red fruits. A three grade color scale (green, orange, and red) was employed to evaluate tomato color.

2.1.4 Amount

The harvested fruits were chopped and 1 g fresh weight (FW) of the pieces was put in a 2-ml tube with a 5 mm of Zirconia bead to be used for metabolomics profiling and 3 g was saved for ELISA and qRT-PCR assays.

GC-MS an equivalent of 0.6 μg and 6 μg of the derivatized samples were injected.

LC-MS an equivalence of 125 μg was injected.

CE-MS an equivalence of 14 μg was injected.

2.1.5 Growth condition

Seedlings of Solanum lycopersicum were potted in 1/2000 a Wagner pot containing compost soil (Kureha, Tokyo, Japan) for the soil experiment. Seeds were sown in 5 cm × 5 cm × 5 cm (height × length × width) rockwool cubes and grown in a hydroponics system (565 mg l^{-1} NO_3^-, 15.7 mg l^{-1} NH_4^+, 202.2 mg l^{-1} PO_3^-, 218.4 mg l^{-1} K^+, 19.9 mg l^{-1} Mg_2^+, 95.0 mg l^{-1} Ca_2^+ and micronutrients) in an environmentally controlled growth room at 25 °C/20 °C (light/dark) and 600 ppm CO_2 concentration with a light/dark cycle of 16 h/8 h for the hydroponic culture (HC) experiment. Seedlings were placed in a netted-greenhouse located at the Gene Research Center in University of Tsukuba.

2.1.6 Experimental condition

Same as growth conditions.

2.1.7 Sampling and sampling date

The fruits were harvested in spring (a pilot and HC experiments) and late summer (the soil experiment) in 2006, 2008, and 2009.

2.1.8 Metabolism quenching method

All samples were frozen within 30 s after sampling in liquid nitrogen. The frozen samples were lyophilized.

2.2 Chemical analysis meta data

2.2.1 Sample processing and extraction

The lyophilized sample in a 2 ml tube was frozen and then homogenized with a 5 mm of zirconia bead by a Mixer Mill (Retsch, Haan, Germany) at 20 Hz for 1 min. Five mg dry weight (DW) of the lyophilized samples were weighed for GC-MS and LC-MS analyses, while 25 mg DW of the samples for CE-MS analysis.

Extraction and derivatization for GC-MS: Each sample was extracted with a concentration of 2.5 mg DW of tissues per ml extraction medium (methanol / chloroform/water [3:1:1 v/v/v]) containing 10 stable isotope reference compounds:

- [$^{2}H_4$]-succinic acid,
- [$^{13}C_5$,^{15}N]-glutamic acid,
- [$^{2}H_7$]-cholesterol,
- [$^{13}C_3$]-myristic acid,
- [$^{13}C_5$]-proline,
- [$^{13}C_{12}$]-sucrose,
- [$^{13}C_4$]-hexadecanoic acid,
- [$^{2}H_4$]-1,4-butanediamine,
- [$^{2}H_6$]-2-hydoxybenzoic acid and
- [$^{13}C_6$]-glucose

using a Retsch mixer mill MM310 at a frequency of 30 Hz for 3 min at 4°C. Each isotope compound was adjusted to a final concentration of 15 ng μl^{-1} for each 1-µl injection. After centrifugation for 5 min at 15,100 × g, a 200-µl aliquot of the supernatant was drawn and transferred into a glass insert vial. The extracts were evaporated to dryness in an SPD2010 SpeedVac® concentrator from ThermoSavant (Thermo electron corporation, Waltham, MA, USA). For methoximation, 30 µl of methoxyamine hydrochloride (20 mg/ml in pyridine) was added to the sample. After 24 h of derivatization at room temperature, the sample was trimethylsilylated for 1 h using 30 µl of MSTFA with 1% TMCS at 37°C with shaking. Thirty µl of n-heptane was added following silylation. All the derivatization steps were performed in the vacuum glove box VSC-100 (Sanplatec, Japan) filled with 99.9995% (G3 grade) of dry nitrogen.

For methoximation, 30 µl of methoxyamine hydrochloride (20 mg ml^{-1} in pyridine) was added to the sample. After 24 h of derivatization at room temperature, the sample was trimethylsilylated for 1 h using 30 µl of MSTFA with 1% TMCS at 37°C with shaking. Thirty μl of n-heptane was added following silylation. All the derivatization steps were performed in the vacuum glove box VSC-100 (Sanplatec, Japan) filled with 99.9995% (G3 grade) of dry nitrogen.

Extraction for LC-MS Five mg DW per 150 μl of extraction medium (methanol/water [2:5 v/v] with reference compounds [0.5 mg l^{-1} flavonol-2'-sulfonic acid and 1.0 mg l^{-1} ampicilin]) each sample was used for the extraction of plant material using a Retsch mixer mill MM310 at a frequency of 20 Hz for 5 min at 4°C. After centrifugation for 10 min at 15,000 × g, the supernatant was transferred into a 2 ml tube. Thirty volumes of methanol were added to the tube and then extracted again using the mixer mill at a frequency of 20 Hz for 5 min at 4°C. After centrifugation for 10 min at 15,000 × g, the resulting supernatant was transferred into the tube. Two hundred-μl aliquot of the extracts was filtered using an Oasis® HLB μ-elusion plate (30 μm, Waters Co., Massachusetts, USA). The extracts were evaporated to dryness in an SPD2010 SpeedVac® concentrator from ThermoSavant (Thermo electron corporation, Waltham, MA, USA). The extracts were dissolved by 160 μl of 20% aqueous methanol containing 0.5 mg l^{-1} lidocaine and d-camphor sulfonic acid.

Extraction for CE-MS Each sample was extracted in 200 volumes of methanol containing 8μM of two reference compounds (methionine sulfone for cation and camphor 10-sulfonic acid for anion analyses) using a Retsch mixer mill MM310 at a frequency of 27 Hz for 1 min. The extracts were then centrifuged at 20,400 × g for 3 min at 4 °C. Five hundred-μl aliquot of the supernatant was transferred into a tube. Five hundred μl of chloroform and 200 μl of water was added into the tube to perform liquid-liquid distribution. The upper layer was evaporated for 30 min at 45°C by a centrifugal concentrator to obtain two layers. For removing high-molecular-weight compounds such as oligo-sugars, the upper layer was centrifugally filtered through a Millipore 5-kDa cutoff filter at 9,100 g for 120 min at 4°C. The filtrate was dried for 120 min by a centrifugal concentrator. The residue was dissolved into 20 μl of water containing 200 μM of internal standards (3-aminopyrrolidine for cation and trimesic acid for anion analyses) that were used for compensation of migration time in the peak annotation step.

2.2.2 GC-TOF/MS conditions

One microliter of each sample was injected in the splitless mode by an CTC CombiPAL autosampler (CTC analytics, Zwin-gen, Switzerland) into an Agilent 6890N gas chromatograph (Agilent Technologies, Wilmingston, USA) equipped with a 30 m × 0.25 mm inner diameter fused-silica capillary column with a chemically bound 0.25-μl film Rtx-5 Sil MS stationary phase (RESTEK, Bellefonte, USA) for metabolome analysis.

Helium was used as the carrier gas at a constant flow rate of 1 ml min^{-1}. The temperature program for metabolome analysis started with a 2-min isothermal step at 80 ◦C and this was followed by temperature ramping at 30 ◦C to a final temperature of 320 ◦C, which was maintained for 3.5 min. The transfer line and the ion source temperatures were 250 and 200 °C, respectively. Ions were generated by a 70-eV electron beam at an ionization current of 2.0 mA. The acceleration

voltage was turned on after a solvent delay of 273 s. Data acquisition was performed on a Pegasus IV TOF mass spectrometer (LECO, St. Joseph, MI, USA) with an acquisition rate of 30 spectra s^{-1} in the mass range of a mass-to-charge ratio of m/z = 60–800.

Alkane standard mixtures (C8–C20 and C21–C40) were purchased from Sigma–Aldrich (Tokyo, Japan) and were used for calculating the retention index (RI) [7, 8]. The normalized response for the calculation of the signal intensity of each metabolite from the mass-detector response was obtained by each selected ion current that was unique in each metabolite MS spectrum to normalize the peak response. For quality control, we injected methylstearate in every 6 samples. Data was normalized using the CCMN algorithm [9].

2.2.3 LC-TOF/MS conditions

After filtration of the extracts (Ultrafree-MC, 0.2 μm pore size; Millipore), the sample extracts (5 μl) were analyzed using an LC-MS system equipped with an electrospray ionization (ESI) interface (HPLC, Waters Acquity UPLC system; MS, Waters Q-Tof Premier). The analytical conditions were as follows. HPLC: column, Acquity bridged ethyl hybrid (BEH) C18 (pore size 1.7 μ m, length 2.0 × 100 mm, Waters); solvent system, acetonitrile (0.1% formic acid):water (0.1% formic acid); gradient program, 1 : 99 v/v at 0 min, 1 : 99 v/v at 0.1 min, 99.5 : 0.5 at 15.5 min, 99.5 : 0.5 at 17.0 min, 1 : 99 v/v at 17.1 min and 1 : 99 at 20 min; flow rate, 0.3 ml min−1; temperature, 38°C; MS detection: capillary voltage, +3.0 keV; cone voltage, 23 V for positive mode and 35 V for negative mode; source temperature, 120°C; desolvation temperature, 450°C; cone gas flow, 50 l h−1; desolvation gas flow, 800 l/ h; collision energy, 2 V for positive mode and 5 V for negative mode ; detection mode, scan (m/z 100–2000; dwell time 0.45 sec; interscan delay 0.05 sec, centroid). The scans were repeated for 19.5 min in a single run. The data were recorded using MassLynx version 4.1 software (Waters).

2.2.4 CE-TOF/MS conditions

CE-TOF MS instruments: All CE-TOFMS experiments were performed using an Agilent CE capillary electrophoresis system (Agilent Technologies, Waldbronn, Germany), an Agilent G3250AA LC/MSD TOF system (Agilent Technologies, Palo Alto, CA), an Agilent 1100 series binary HPLC pump, and the G1603A Agilent CE-MS adapter and G1607A Agilent CE-ESI-MS sprayer kit. The G2201AA Agilent ChemStation software for CE and the Analyst QS software for TOFMS were used.

Separation column and electrolytes: Separations were carried out using a fused silica capillary (50 μm i.d. x 100 cm total length) filled with 1 M formic acid for cation analyses or with 20 mM ammonium formate (pH 10.0) for anion analyses as the electrolyte. The capillary temperature was maintained at 20 °C.

Sample injection: The sample solutions were injected at 50 mbar for 15 sec (15 nL). The sample tray was cooled below 4 °C.

Separation parameters: Prior to each run the capillary was flushed with electrolyte for 5 min. The applied voltage for separation was set at 30 kV. Fifty percent (v/v) methanol/water containing 0.5 μM reserpine was delivered as the sheath liquid at 10 μL/min.

Ionization: ESI-TOFMS was conducted in the positive ion mode for cation analyses or in the negative ion mode for anion analyses, and the capillary voltage was set at 4 kV.

Dry gas condition: A flow rate of heated dry nitrogen gas (heater temperature 300 °C) was maintained at 10 psig.

Voltage settings in TOF/MS: The fragmentor, skimmer, and Oct RFV voltage were set at 110V, 50V, and 160V for cation analyses or at 120V, 60V, and 220V for anion analyses, respectively.

Mass calibration: Automatic recalibration of each acquired spectrum was performed using reference masses of reference standards. The methanol dimer ion ($[2M+H]^+$, m/z = 65.0597) and reserpine ([M+H]+, m/z = 609.2806) for cation analyses or the formic acid dimer ion ([2M-H]-, m/z = 91.0037) and reserpine ($[M-H]^-$, m/z = 607.2661) for anion analyses provided the lock mass for exact mass measurements.

Mass data acquirement: Exact mass data were acquired at a rate of 1.5 cycles/sec over a 50-1000 m/z range.

Quality control: In an every single sequence analysis (maximum 36 samples) on our CE-MS system, we analyzed the standard compound mixture at the first and the end of sample analyses. The detected peak area of standard compound mixture was checked in point of reproducible sensitivity. Standard compound mixture composed of major detectable metabolites including amino acids and organic acids, and this mixture was newly prepared at least once a half year. In all analyses in this study, there were no differences in the sensitivity of standard compounds mixture.

2.2.5 Data processing

GC-MS Nonprocessed MS data from GC-TOF/MS analysis were exported in NetCDF format generated by chromatography processing and mass spectral deconvolutionsoftware, Leco ChromaTOF version 3.22 (LECO, St. Joseph, MI, USA) to MATLAB 6.5 (Mathworks, Natick, MA, USA), where all data-pretreatment procedures, such as smoothing, alignment, time-window setting, and H-MCR, were carried out [10]. The resolved MS spectra were matched against reference mass spectra using the NIST mass spectral search program for the NIST/EPA/NIH mass spectral library (version 2.0) and our custom software for peak annotation written in JAVA. Peaks were identified or annotated based on RIs and the reference mass spectra comparison to the Golm Metabolome Database (GMD) released from CSB.DB[1] [11]] and our in-house spectral library. The metabolites were identified by comparison with RIs from the library databases (GMD and our own library) and with those of authentic standards, and the metabolites were defined as annotated metabolites on comparison with mass spectra and RIs from these two libraries.

LC-MS The profiling data files recorded in the MassLynx format (raw) were converted to the NetCDF format using the DataBridge function of MassLynx 4.1. From the set of NetCDF data files, the data matrix was generated using the MetAlign software (De Vos et al., 2007). By using this procedure, the data matrixes with unit mass data were generated. The data matrices were processed using in-house software written in Perl/Tk. The original peak intensity values were divided with that of the internal standards (lidocaine at m/z 235 [M + H]+ and (–)-camphor-10-sulfonic acid at m/z 231 [M – H]– for the positive and negative ion modes, respectively) determined in the same samples to normalize the peak intensity values among the metabolic profile data.

CE-MS An original data file (.wiff) was converted to an unique binary file (.kiff) using in-house software (nondisclosure). Peak picking and alignment were performed using the another in-house software (nondisclosure), peaks were picked and aligned among samples automatically. By contrast with the detected m/z and migration time values of standard compounds including internal standards, peaks were annotated automatically using the same software. For normalization, the individual area of the detected peaks was divided by the peak area of the internal reference standards. Based on the calibration curves for standard compounds, peak area values were converted into values corresponding to amounts.

[1] http://csbdb.mpimp-golm.mpg.de/csbdb/gmd/msri/gmd_msri.html

Supporting References

[1] Johnson WE, Li C, Rabinovic A (2007) Adjusting batch effects in microarray expression data using empirical Bayes methods. Biostatistics 8: 118–127.

[2] Hothorn LA, Oberdoerfer R (2006) Statistical analysis used in the nutritional assessment of novel food using the proof of safety. Regul Toxicol Pharmacol 44: 125–135.

[3] Tempelman RJ (2004) Experimental design and statistical methods for classical and bioequivalence hypothesis testing with an application to dairy nutrition studies. J Anim Sci 82 E-Suppl: E162–E172.

[4] Bylesjö M, Rantalainen M, Cloarec O, Nicholson JK, Holmes E, et al. (2006) OPLS discriminant analysis: combining the strengths of PLS-DA and SIMCA classification. J Chemom 20: 341-351.

[5] Stacklies W, Redestig H, Scholz M, Walther D, Selbig J (2007) pcaMethods – a Bioconductor package providing PCA methods for incomplete data. Bioinformatics 23: 1164–1167.

[6] Bais P, Moon SM, He K, Leitao R, Dreher K, et al. (2010) PlantMetabolomics.org: a web portal for plant metabolomics experiments. Plant Physiol 152: 1807–1816.

[7] Schauer N, Steinhauser D, Strelkov S, Schomburg D, Allison G, et al. (2005) GC-MS libraries for the rapid identification of metabolites in complex biological samples. FEBS Lett 579: 1332–1337.

[8] Wagner C, Sefkow M, Kopka J (2003) Construction and application of a mass spectral and retention time index database generated from plant GC/EI-TOF-MS metabolite profiles. Phytochemistry 62: 887–900.

[9] Redestig H, Fukushima A, Stenlund H, Moritz T, Arita M, et al. (2009) Compensation for systematic cross-contribution improves normalization of mass spectrometry based metabolomics data. Anal Chem 81: 7974-7980.

[10] Jonsson P, Johansson AI, Gullberg J, Trygg J, A J, et al. (2005) High-throughput data analysis for detecting and identifying differences between samples in GC/MS-based metabolomic analyses. Anal Chem 77: 5635–5642.

[11] Kopka J, Schauer N, Krueger S, Birkemeyer C, Usadel B, et al. (2005) GMD@CSB.DB: the Golm Metabolome Database. Bioinformatics 21: 1635–1638.

Supporting Table S 1: **Overview of the design of three expierments for SE evaulation of miraculin over-expressing tomato.**

	Year 1	Year 2	Year 3
Harvesting season	Spring	Spring	Summer
Control	Moneymaker	Moneymaker	Moneymaker
Transgene	Miraculin-OX	Miraculin-OX	Miraculin-OX
Number of alleles	2	2	2
Transgene llines	56B, 7C	56B, 7C	56B, 7C
Number of traditional cultivars	0	5	0
Number of fruits / genotype	3	6 to 8	15 + 15 (Moneymaker, more water + less water), 29 + 28 (56B, mw + lw), 18 + 28 (7C, mw + lw)
Number of plants / genotype	3	6 to 8	5 + 4 (Moneymaker, more water + less water), 5 + 6 (56B, mw + lw), 5 + 4 (7C, mw + lw)
Tissue	Fruit	Fruit	Fruit
Stage	Red	Green and Red	Red
Truss numbers	2	2	2 to 4
Growth condition	Soil and HC	HC	Soil
Watering	No	No	More water and less water

Supporting Table S 2: **Complexities of the used OPLS-DA models.** n_A shows the number of predictive models, n_{A_O} the number of orthogonal components. Performance was caluclated by five-fold cross-validation.

Model	n_A	n_{A_O}	Performance in CV (accuracy) 3
All genotypes on HC	6	2	See Figure 3.
Moneymaker versus 56B, HC	1	1	0.7
Moneymaker vertsus 7C, HC	1	1	0.73
Moneymaker versus 56B, Soil	1	1	0.92
Moneymaker versus 7C, Soil	1	2	0.92

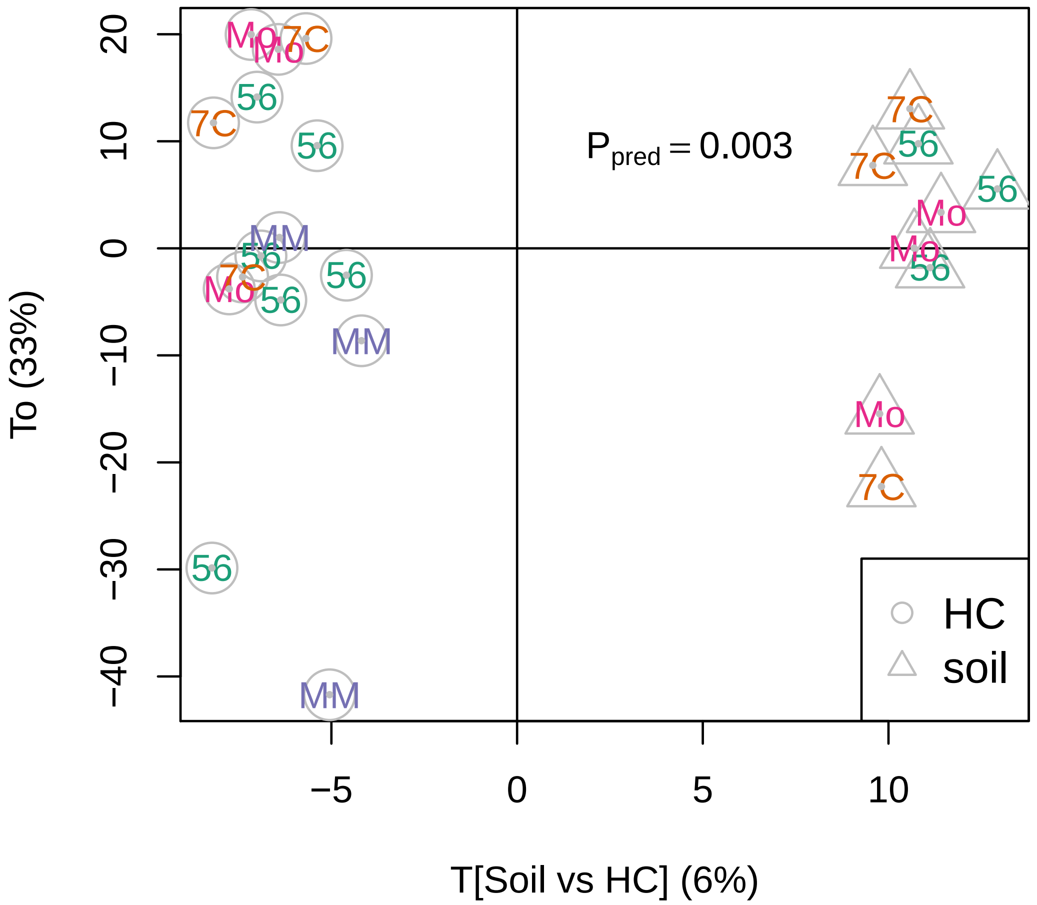

Supporting Figure S 1: **Discrimination analysis of the data from the pilot experiment using OPLS-DA.** Percentages in axis labels indicate the ratio of the total variance that is accounted for. The observed $P_{\text{Pred}} = 0.003$ indicates that observed classification accuracy under cross-validation, (accuracy 0.83) is significanly better than observed with randomized data. The two growth conditions account for ca 6% of the total variance. *Abbreviations: MM, Moneymaker; 56, 56B; HC, hydroponic culture solution.*

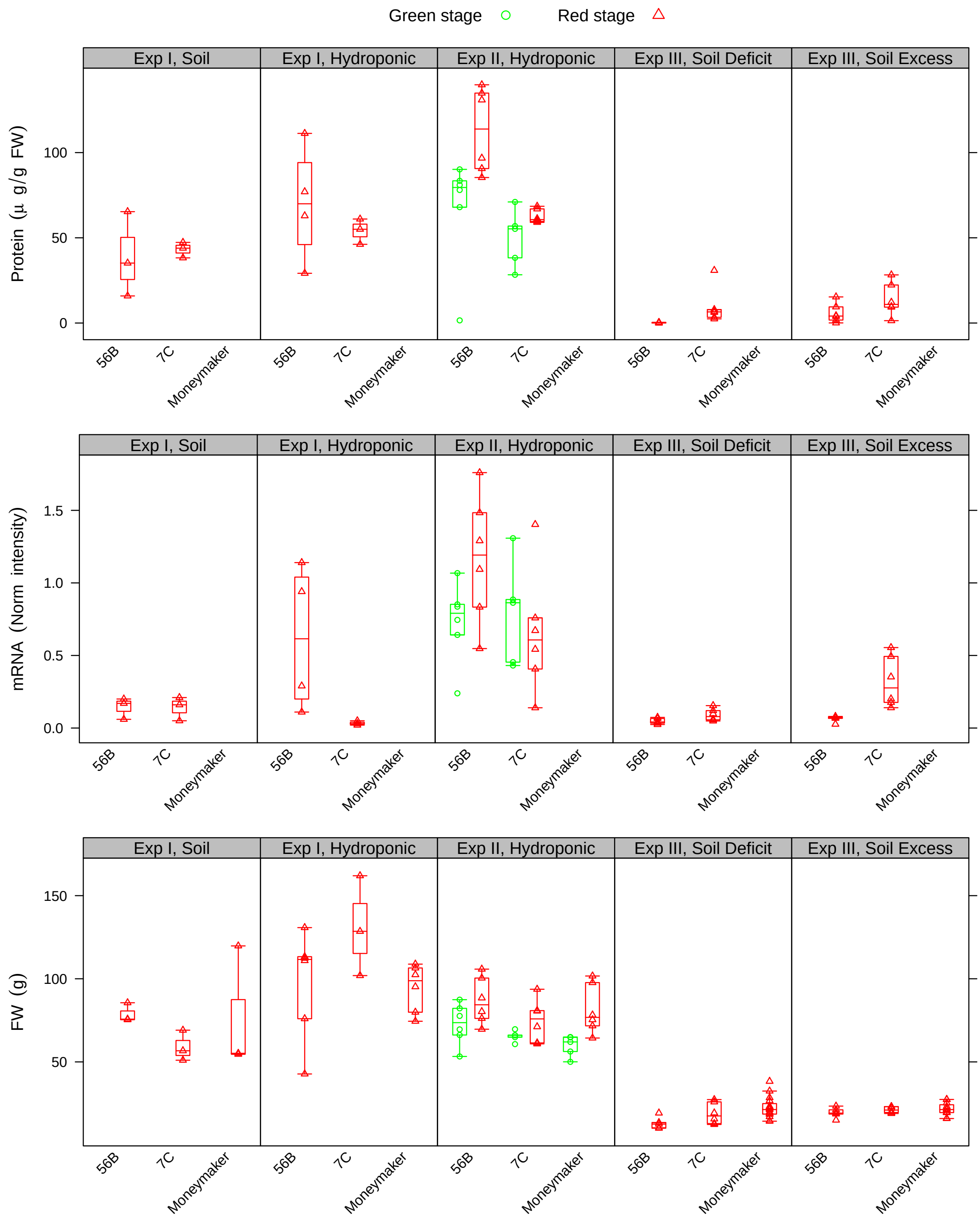

Supporting Figure S 2: **Fresh weight and miraculin mRNA and protein accumulation in the different data sets.** Protein content was measured using ELISA and mRNA using qRT-PCR.

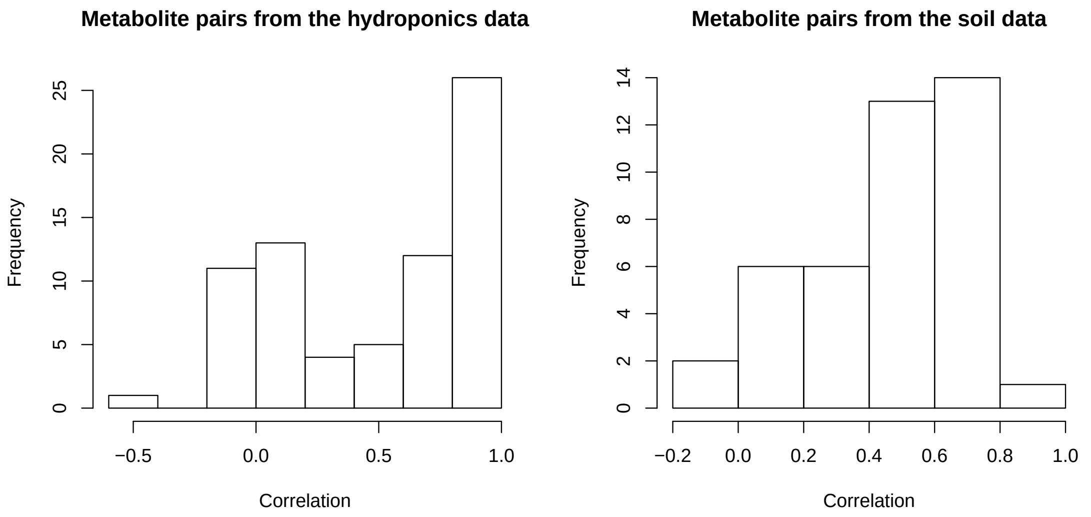

Supporting Figure S 3: **Correlation of multiply detected metabolites.** Shown are histograms of correlation coefficients between pairs of peaks representing the same metabolite in the hydroponics and soil data respectively.

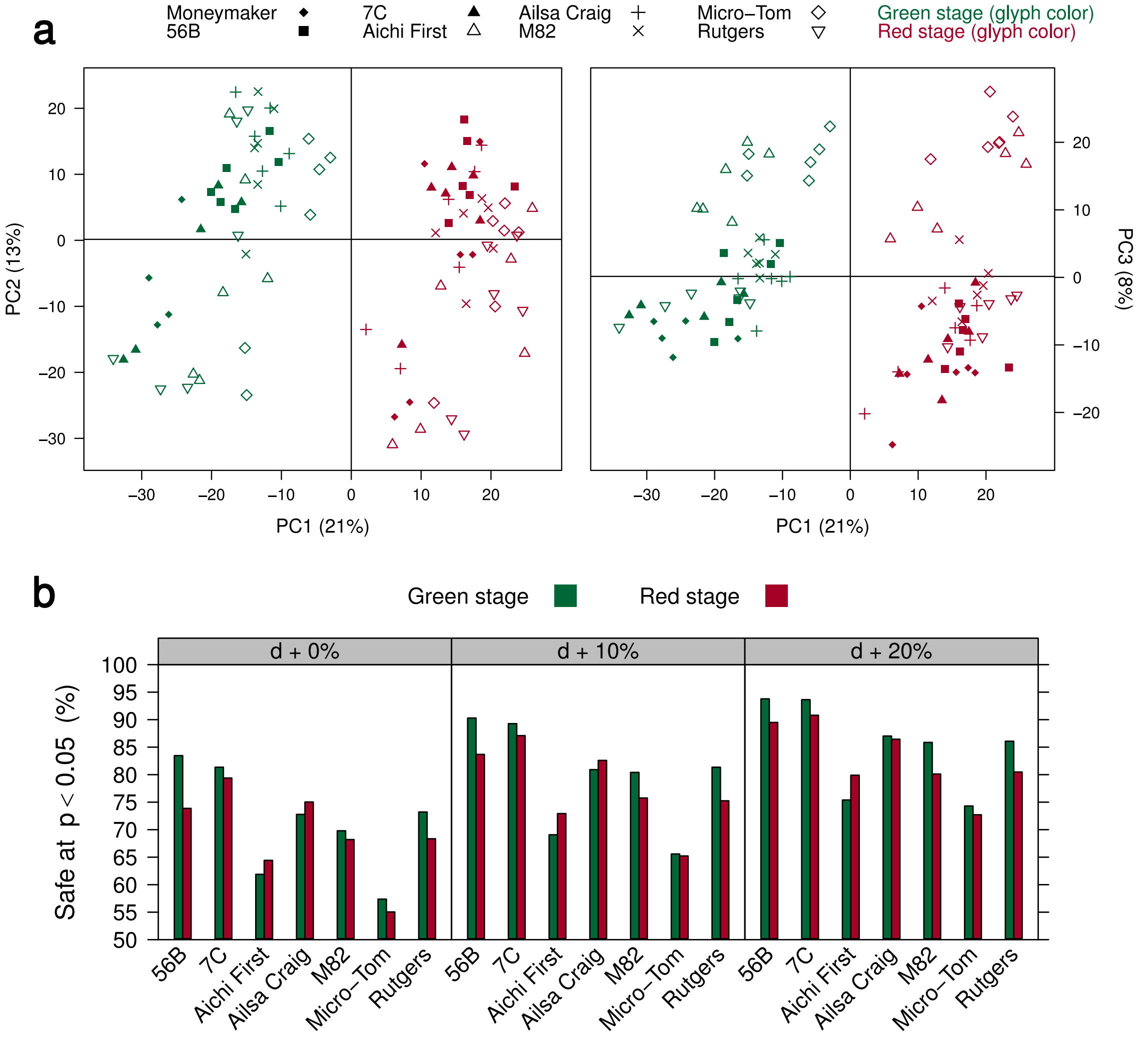

Supporting Figure S 4: **Overview of the metabolite data from the hydroponics experiment.** (a) PCA of the full (1639 peaks) summarized data set of the hydroponics experiment. PC1 is correlated to the development stage. PC2 is of unknown biological, unrelated to all known experimental factors. PC3 is correlated to the genotype. (b) The percentages of the peaks for which non-safety could be rejected at $p < 0.05$ when using asymmetric thresholds for the acceptable limits of deviation from the control and allowing for 0, 10 and 20% deviation from the upper or lower limit.

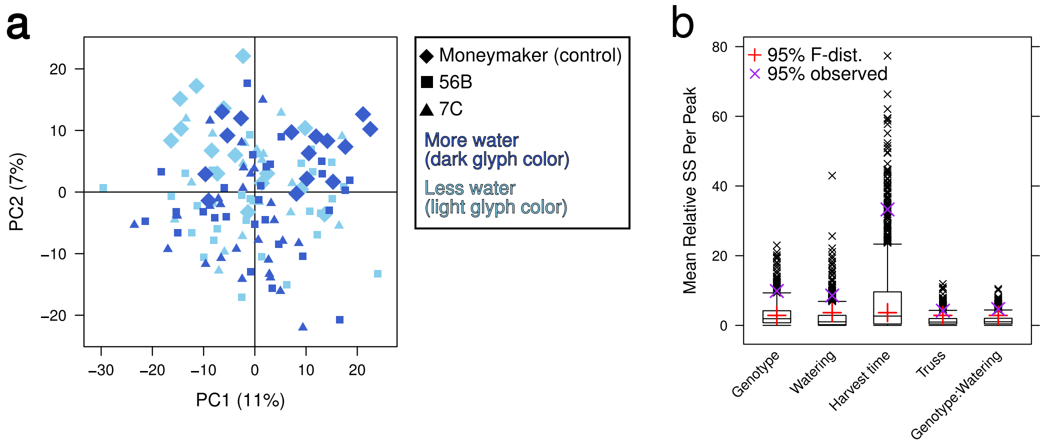

Supporting Figure S 5: **Overview of the metabolite data from the hydroponics experiment.** (a) Overview PCA of the soil experiment. Slight grouping of the unmodified plants can be seen. (b) Contribution of variance in the soil experiment. Peaks above the 95th F-test significant percentile indicate that points show significance at 0.05 significance level (expected 5% false positives). Factors where the empirical percentile are far above the F-test percentile appear to have a genuine effect of the metabolite abundance.

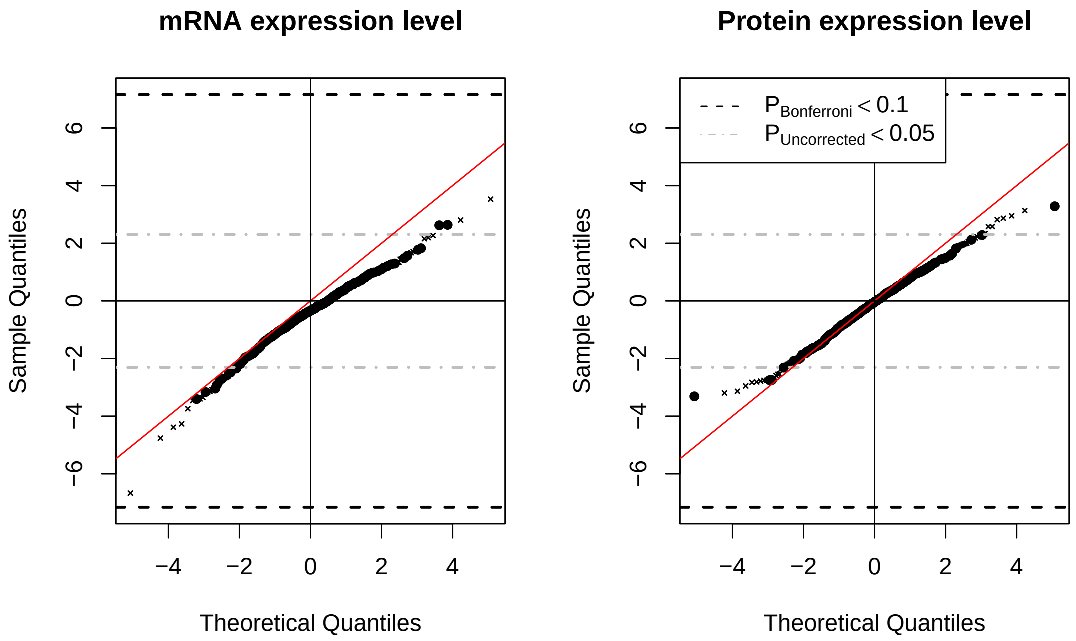

Supporting Figure S 6: **Investigating the presence of metabolite-miraculin correlations.** Shown is the observed and theoretical null-hypothesis quantiles of t-values for the significance of the correlation between miraculin expression levels (protein and mRNA) and each metabolite peak in 7C in the soil experiment.

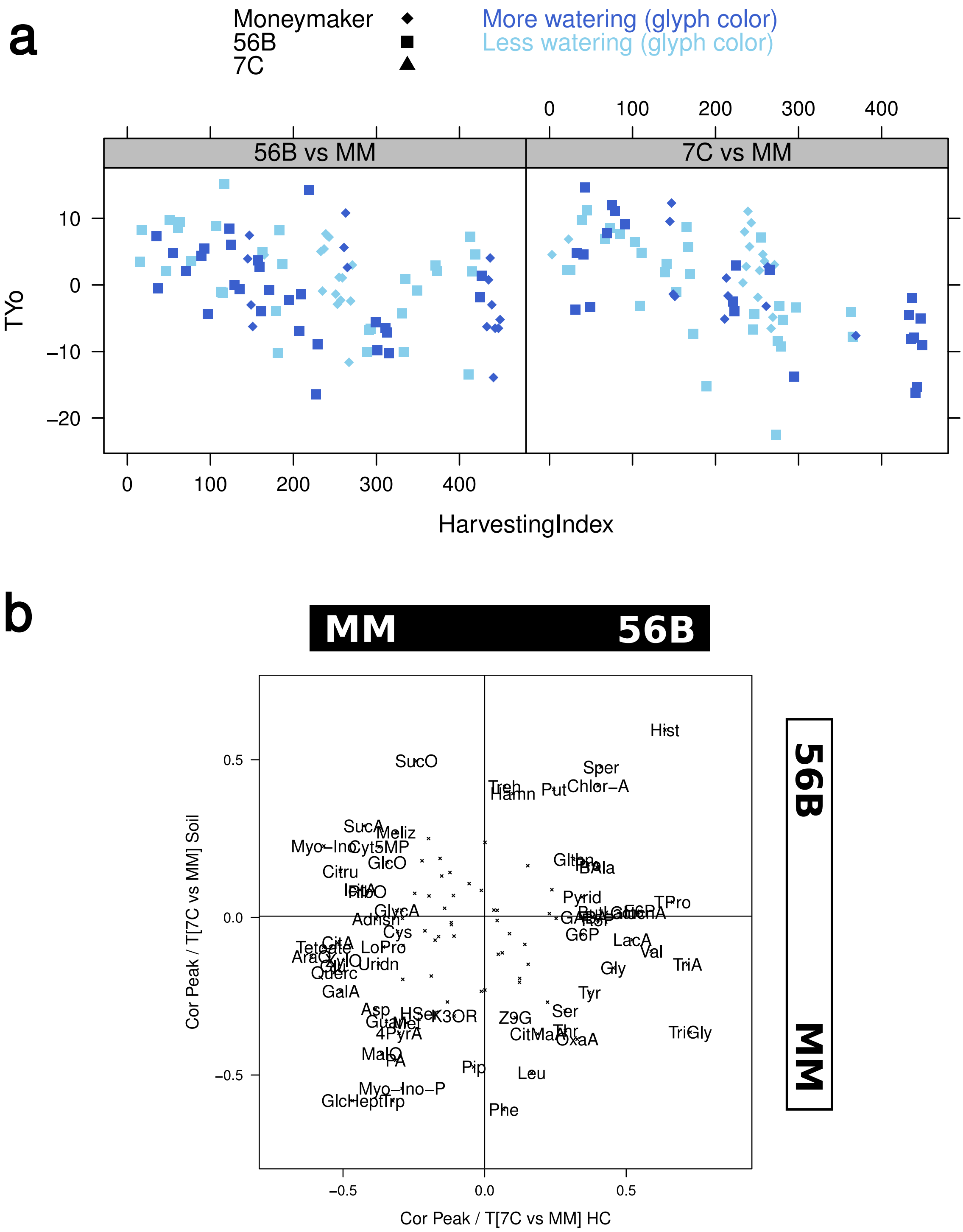

Supporting Figure S 7: **Examining the OPLS-DA models from the soil experiment.** (a) The genotype orthogonal components in Figure 5a-b, T_O, is negatively correlated to harvesting index (the chronological order in which the samples were harvested). (b) Scatter plot of the loadings of the metabolites used to discriminate 56B and Moneymaker in the HC (black bar) and the soil experiment (white bar). There is no significant overlap between the two responses.

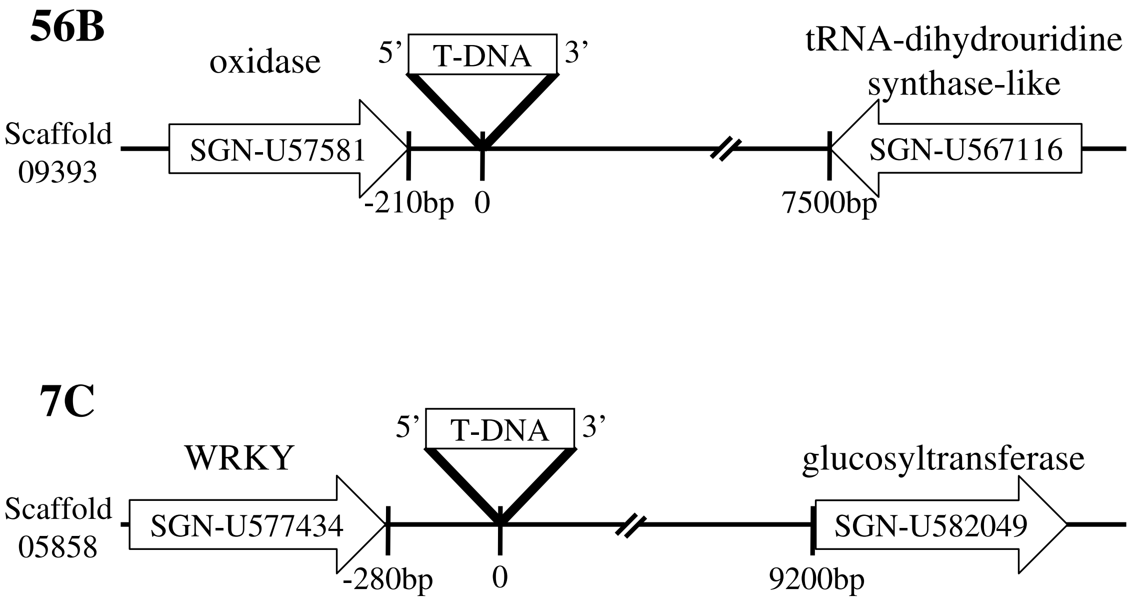

Supporting Figure S 8: **The positions of the inserts as verified by DNA sequencing.**

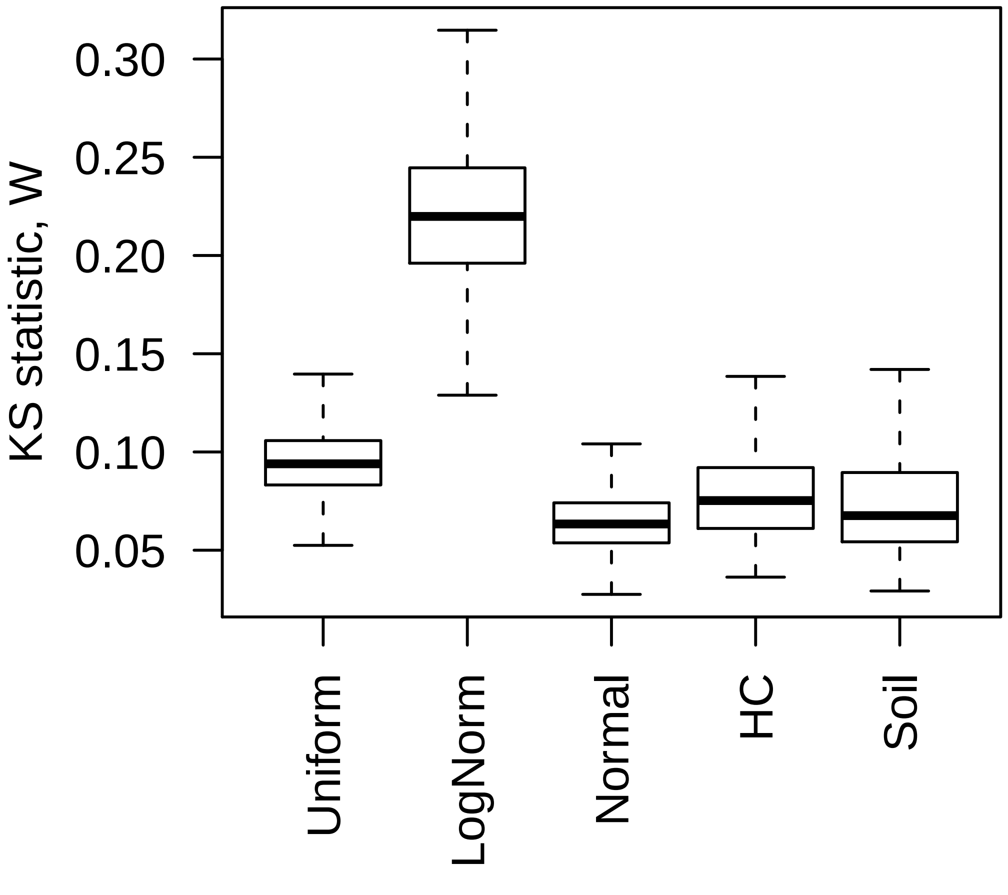

Supporting Figure S 9: **Examining the normality of residuals for the HC and the soil experiments.** Shown are the distribution of the KS test for normality of the residuals for each peak. KS statistics for random deviates from perfect uniform, log-normal and normal distributions are shown for comparison. A high KS statistic indicates a deviation from the normal distribution.
